# The C9orf72-interacting protein Smcr8 is a negative regulator of autoimmunity and lysosomal exocytosis

**DOI:** 10.1101/gad.313932.118

**Published:** 2018-07-01

**Authors:** Yingying Zhang, Aaron Burberry, Jin-Yuan Wang, Jackson Sandoe, Sulagna Ghosh, Namrata D. Udeshi, Tanya Svinkina, Daniel A. Mordes, Joanie Mok, Maura Charlton, Quan-Zhen Li, Steven A. Carr, Kevin Eggan

**Affiliations:** 1 Department of Stem Cell and Regenerative Biology, Harvard University, Cambridge, Massachusetts 02138, USA;; 2 Department of Molecular and Cellular Biology, Harvard University, Cambridge, Massachusetts 02138, USA;; 3 Stanley Center for Psychiatric Research, Broad Institute of Massachusetts Institute of Technology and Harvard, Cambridge, Massachusetts 02142, USA;; 4 Proteomics Platform, Broad Institute of MIT and Harvard, Cambridge, Massachusetts 02142, USA;; 5 Department of Pathology, Massachusetts General Hospital, Boston, Massachusetts 02114, USA;; 6 Department of Immunology, University of Texas Southwestern Medical Center, Dallas, Texas 75390, USA;; 7 Department of Internal Medicine, University of Texas Southwestern Medical Center, Dallas, Texas 75390, USA

**Keywords:** amyotrophic lateral sclerosis (ALS), C9ORF72, SMCR8, autophagy, lysosome, autoimmunity

## Abstract

While a mutation in *C9ORF72* is the most common genetic contributor to amyotrophic lateral sclerosis (ALS), much remains to be learned concerning the function of the protein normally encoded at this locus. To elaborate further on functions for C9ORF72, we used quantitative mass spectrometry-based proteomics to identify interacting proteins in motor neurons and found that its long isoform complexes with and stabilizes SMCR8, which further enables interaction with WDR41. To study the organismal and cellular functions for this tripartite complex, we generated *Smcr8* loss-of-function mutant mice and found that they developed phenotypes also observed in *C9orf72* loss-of-function animals, including autoimmunity. Along with a loss of tolerance for many nervous system autoantigens, we found increased lysosomal exocytosis in *Smcr8* mutant macrophages. In addition to elevated surface Lamp1 (lysosome-associated membrane protein 1) expression, we also observed enhanced secretion of lysosomal components—phenotypes that we subsequently observed in *C9orf72* loss-of-function macrophages. Overall, our findings demonstrate that C9ORF72 and SMCR8 have interdependent functions in suppressing autoimmunity as well as negatively regulating lysosomal exocytosis—processes of potential importance to ALS.

## Abstract (precis)

A mutation in C9ORF72 is the most common genetic contributor to ALS. Zhang et al. found that C9ORF72's long isoform complexes with and stabilizes SMCR8. Smcr8 loss-of-function mutant mice exhibit a loss of tolerance for many nervous system autoantigens and increased lysosomal exocytosis in mutant macrophages.

Amyotrophic lateral sclerosis (ALS) is a progressive and lethal neurodegenerative disease that affects both the upper and lower motor neurons (Robberecht and Philips 2013). A GGGGCC repeat expansion mutation in *C9ORF72* is the most commonly known genetic cause of ALS and frontotemporal lobar degeneration (FTLD/FTD) (DeJesus-Hernandez et al. 2011; Renton et al. 2011). In North America, this mutation is found in 40% of familial ALS cases and 7% of sporadic ALS cases (Majounie et al. 2012). The repeat expansion leads to the formation of RNA foci (Donnelly et al. 2013) and dipeptide repeat protein aggregates (Gendron et al. 2013; Mori et al. 2013a, b), both of which can exhibit toxicity to neurons and may therefore be contributing to disease progression (DeJesus-Hernandez et al. 2011; Donnelly et al. 2013; Gendron et al. 2013; Mori et al. 2013a,b; Kwon et al. 2014; May et al. 2014). In addition, a significant reduction of the gene product normally encoded at *C9ORF72* has been observed in ALS patients both with and without the *C9ORF72* repeat expansion mutation (Belzil et al. 2013; Ciura et al. 2013; Xi et al. 2013). Intriguingly, higher mRNA levels of *C9ORF72* transcript variant 1 (NM_145005.6) have been associated with a longer survival after ALS onset (van Blitterswijk et al. 2015), consistent with the notion that insufficiency of this gene product may play a role in disease. However, the extent to which each of the three ramifications of the repeat expansion described thus far contribute to or collaborate to drive disease in patients remains unresolved (Shi et al. 2018).

The consequence of *C9ORF72* haploinsufficiency in ALS patients could be better understood if the function of the protein normally encoded at this locus could be clarified. The protein sequence of C9ORF72 shares homology with the DENN (differentially expressed in normal and neoplasia) family of proteins and therefore has been proposed to function as a GDP–GTP exchange factor (GEF) for Rab GTPases and may regulate vesicular trafficking (Zhang et al. 2012; Levine et al. 2013). Protein–protein interaction studies showed that C9ORF72–SMCR8–WDR41 formed a complex (Amick et al. 2016; Ciura et al. 2016; Sellier et al. 2016; Sullivan et al. 2016; Ugolino et al. 2016; Yang et al. 2016; Corbier and Sellier 2017) that had GEF activity for Rab8a and Rab39b (Sellier et al. 2016). In addition, the C9ORF72 protein complex also interacted with ULK1–FIP200–ATG13, raising the possibility that C9ORF72 could function in the autolysosome pathway (Sullivan et al. 2016; Webster et al. 2016; Yang et al. 2016; Jung et al. 2017). Consistent with this idea, lysosomal localization of C9orf72 and Smcr8 have been reported (Amick et al. 2016), and deficiency of these proteins altered the expression of autophagy markers p62 and LC3 (Sellier et al. 2016; Ugolino et al. 2016; Yang et al. 2016). However, the respective roles of C9ORF72 and SMCR8 in these processes have not been resolved, with some studies indicating that this protein complex positively regulates autophagy (Sellier et al. 2016) and others arguing that it has a negative effect on the pathway (Ugolino et al. 2016). Adding ambiguity, it has also been proposed that C9orf72 and Smcr8 could play opposing roles in the autophagy pathway (Yang et al. 2016).

Previously, we and others found that loss-of-function mutations in the mouse *C9orf72* ortholog resulted in autoimmunity (Atanasio et al. 2016; Burberry et al. 2016; O'Rourke et al. 2016). Abnormal p62 processing and accumulation of lysosome-associated membrane protein 1 (Lamp1)-positive vesicles have also been observed in macrophages from these mutant mice (O'Rourke et al. 2016), providing in vivo evidence supporting a role of C9orf72 in the autolysosome pathway. However, several major questions remain to be answered: Are there additional unknown C9orf72-interacting proteins that can be readily identified? Does Smcr8 share in vivo functions with C9orf72? Finally, what alterations in the autolysosome pathway could be credible causes of the autoimmunity seen in mutant animals?

We report here an alternative cellular system that allowed us to perform cell type-dependent C9ORF72 protein–protein interaction studies by quantitative mass spectrometry-based proteomics. Using this system, we replicated the finding that C9ORF72 can form a complex with SMCR8 and WDR41 in both human stem cells and human spinal motor neurons derived from stem cells. Coexpression studies led to the surprising finding that C9orf72 and Smcr8 mutually stabilize each other. In addition, we produced *Smcr8* loss-of-function mutant mice and found that they exhibited central phenotypes observed in *C9orf72* mutant animals, including autoimmunity. Further elaborating on the cell biological functions of Smcr8, we found that its loss resulted in increased lysosomal exocytosis, a phenotype subsequently observed in C9orf72-deficient macrophages that is essential for maintaining normal immune system function (Alter et al. 2004; Betts and Koup 2004). In all, our findings provide new compelling evidence that the C9ORF72–SMCR8 protein complex is a negative regulator of autoimmunity, a condition that had been identified recently as often preceding the onset of ALS (Turner et al. 2013; Miller et al. 2016).

## Results

### A system for identifying isoform-specific cell type-dependent C9ORF72-interacting proteins

**Figure 1.**
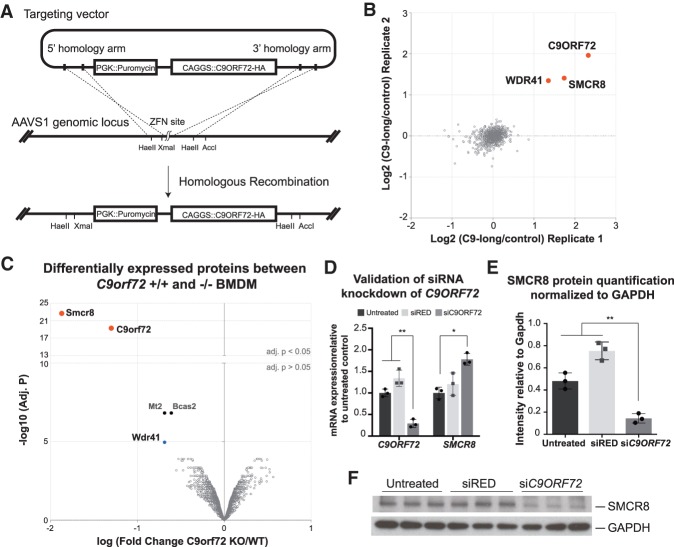
C9ORF72–SMCR8–WDR41 form a protein complex. (*A*) Illustration of the gene-editing strategy for making C9ORF72-HA stable expression hES cell lines. Zinc finger nuclease was used to introduce a double-strand break in the *AAVS1* locus. The puromycin resistance selection gene and *CAGGS*-driven *C9ORF72-HA* expression cassette are stably incorporated into the *AAVS1* locus through homologous recombination. (*B*) Identification of protein coimmunoprecipitated with the C9-long protein isoform by quantitative mass spectrometry. The red dots represent proteins significantly enriched in samples with C9-long-HA expression after anti-HA immunoprecipitation with an adjusted *P*-value < 0.05 by moderated *t*-test. (*C*) Differentially expressed proteins in bone marrow-derived macrophages (BMDMs) from *C9orf72*^+/+^ and *C9orf7*^−/−^ mice by quantitative mass spectrometry. *n* = 2 for each genotype. The red dots represent differentially expressed proteins with an adjusted *P*-value of <0.05 by moderated *t*-test. (*D*–*F*) HEK293 cells were left untreated or were treated with control siRED or siRNA against *C9ORF72* as indicated. (*D*) qPCR validation of *C9ORF72* siRNA knockdown efficiency and *SMCR8* expression level with the indicated treatment. (*E*) Quantification of SMCR8 protein expression normalized to GAPDH by Western blot. For both *D* and *E*, *n* = 3. (*) *P* < 0.05; (**) *P* < 0.01 by two-tailed Student's *t*-test. Error bar represents standard deviation. (*F*) Representative Western blot image for endogenous SMCR8 and GAPDH with the indicated siRNA treatment.

Interactions between C9ORF72, SMCR8, and WDR41 were reported recently (Amick et al. 2016; Sellier et al. 2016; Ugolino et al. 2016; Yang et al. 2016). It is known that C9ORF72 has two different protein isoforms. However, whether the two distinct protein isoforms interact with the same set of proteins has not been carefully addressed. We sought to establish a system that would allow us to study C9ORF72-interacting proteins and function in an isoform- and cell type-specific manner. To this end, we used homologous recombination at the *AAVS1* locus to establish HuES3 human embryonic stem (hES) cell lines stably expressing either the long (C9-long) or short (C9-short) protein isoforms of C9ORF72 fused to a C-terminal hemagglutinin (HA) tag (Fig. 1A; Hockemeyer et al. 2009; Lombardo et al. 2011). Proper *C9ORF72-HA* transgene incorporation into the *AAVS1* locus was confirmed by PCR (Supplemental Fig. 1A). HA-tagged C9ORF72 expression in the engineered cell lines was further validated by quantitative real-time PCR (qPCR) and Western blotting (Supplemental Fig. 1B,C). These polypeptides could be readily immunoprecipitated from preparations of ES cells using anti-HA antibodies (Supplemental Fig. 1C) and were also recognized by established anti-C9ORF72 antibodies (Supplemental Fig. 1D).

To identify C9ORF72-interacting candidates, fractions of hES cells were immunoprecipitated with anti-HA antibodies and analyzed by quantitative liquid chromatography-mass spectrometry with tandem mass tag (TMT) labeling. In the subsequent data analysis, we considered proteins identified by at least two unique peptides. We found that C9ORF72 itself along with SMCR8 and WDR41 were significantly enriched following HA immunoprecipitation from the C9-long-HA cell line compared with the parental nontransgenic control (defined as adjusted *P* < 0.05 by moderated *t*-test with more than twofold enrichment) (Fig. 1B; Supplemental Table 1). Interestingly, these interactions were specific to the long isoform of C9ORF72 and were not detected in the short isoform-expressing sample (Supplemental Fig. 1E,F; Supplemental Table 1). This protein complex was also identified by quantitative mass spectrometry in human motor neurons derived from these hES cell lines (Supplemental Fig. 1G; Supplemental Table 2). Therefore, using our system, we reproducibly established that C9ORF72 interacts with SMCR8 and WDR41 in multiple cellular contexts. We also furthered our understanding of this complex by showing that these interactions are specific to the long protein isoform of C9ORF72 but not the shorter protein isoform. Using a similar approach by comparing proteins enriched after immunoprecipitation from the C9-short-expressing samples, we identified a different set of potential interacting proteins (Supplemental Table 1). However, these candidates could not be readily validated in subsequent immunoprecipitation-Western blot experiments and therefore were not pursued further in this study.

### C9ORF72 stabilizes SMCR8 protein expression

Recently, it had been shown that loss of C9orf72 function leads to inflammatory disease in mice and that changes in macrophage and microglial cells may contribute to this phenotype (Burberry et al. 2016; O'Rourke et al. 2016). In an attempt to understand how loss of C9orf72 function could contribute to these phenotypes, we looked for proteins that were differentially expressed in the bone marrow-derived macrophages (BMDMs) isolated from wild-type (+/+) and *C9orf72* homozygous loss-of-function (−/−) mice using quantitative mass spectrometry. Interestingly, under basal conditions without immune stimulus, two proteins were found to be significantly differentially expressed between *C9orf72*^+/+^ and *C9orf72*^−/−^ macrophages: C9orf72 itself and Smcr8 (Fig. 1C; Supplemental Table 3).

To further investigate how C9ORF72 might regulate the expression of SMCR8, we used RNAi to silence the expression of *C9ORF72* in human embryonic kidney (HEK293) cells and quantified the SMCR8 transcript and protein. Treatment with siRNA against *C9ORF72* was specific and efficient, which resulted in a reduction of 70.8% of *C9ORF72* mRNA expression compared with untreated controls and a 78.4% reduction when compared with control siRNA-treated (siRED) samples. A moderate but significant increase in *SMCR8* transcript level was observed upon knockdown of *C9ORF72* (Fig. 1D, P < 0.05 [*] and P < 0.01 [**] by two-tailed Student's *t*-test). In contrast, Western blot analysis detected a 70.1% reduction of SMCR8 protein level in C9ORF72-deficient samples compared with untreated controls and an 80.9% reduction compared with control siRNA-treated samples (Fig. 1E,F: *P* < 0.01 [**] by two-tailed Student's *t*-test). Moreover, when the SMCR8-GFP fusion protein was transfected into HEK cells, coexpression of C9-long increased the proportion and fluorescence intensity of GFP-positive cells when analyzed by flow cytometry (Supplemental Fig. 2A–C). Such enhancement of SMCR8-GFP stability can also be appreciated by fluorescent confocal imaging (Supplemental Fig. 2D). In contrast, C9-short did not enhance ectopic SMCR8 expression. WDR41, the other protein that we identified to interact with C9-long by coimmunoprecipitation and mass spectrometry, may have a weak stabilizing effect on the SMCR8 protein when analyzed by confocal imaging, an effect that was not appreciated by flow cytometry (Supplemental Fig. 2A–D). Therefore, full-length C9ORF72 not only interacted with but also stabilized the SMCR8 protein.

**Figure 2.**
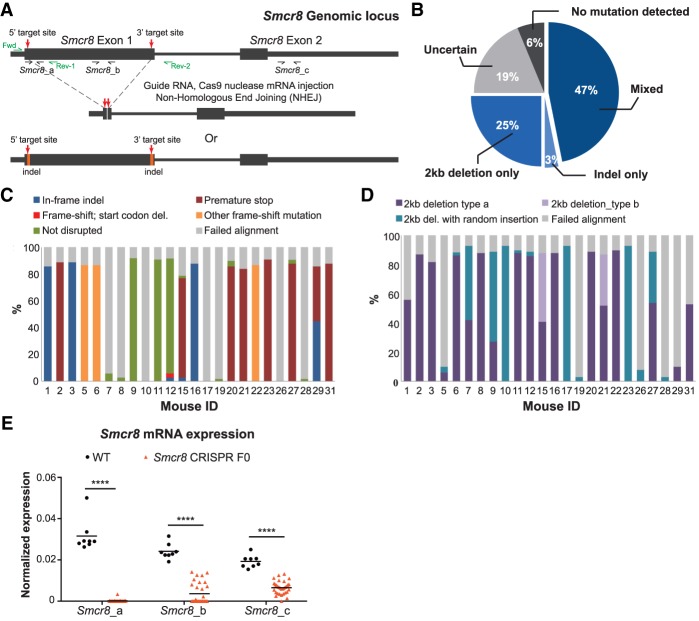
Generation of *Smcr8* loss-of-function mouse model by CRISPR/Cas9. (*A*) A schematic illustration of *Smcr8* knockout strategy. Two CRISPR guides were designed for both the region immediately downstream from the ATG start codon and the region at the end of exon 1 of *Smcr8*. On-target effects of these guides are expected to give rise to two different types of alleles: a large deletion of exon 1 (“2kb del” or “2kb deletion”) or an insertion\deletion (indel) mutation created at each target site. Fwd, Rev-1, and Rev-2 were three primers used for identification of mutant alleles. *Smcr8_a*, *Smcr8_b*, and *Smcr8_c* were three qPCR primer pairs used for examination of *Smcr8* mRNA expression. (*B*) A pie chart representing the relative proportion of *Smcr8* CRISPR F0 mice with each type of mutation, as indicated. The type of mutation was determined by doing PCR using the three genotyping primers indicated in *A* and tail genomic DNA. (*C*,*D*) The frequency of each indicated mutation subtype for each mouse examined by MiSeq. (*C*) Summary of the different types of the 2-kb deletion in exon 1 of the *Smcr8* gene. (*D*) Summary of the various types of indel mutations at the region immediately downstream from the ATG start codon of the *Smcr8* gene. (*E*) *Smcr8* expression in peripheral blood of wild-type (WT) or *Smcr8* CRISPR F0 mice using three different qPCR probe sets as indicated in *A*. *n* = 8 for wild type; *n* = 31 for *Smcr8* CRISPR F0. (****) *P* < 0.0001 by two-way analysis of variance (ANOVA) with Dunnett's multiple comparisons.

### Generation of *Smcr8* knockout mice using CRISPR/Cas9

Given that the C9-long protein isoform stabilized SMCR8, we hypothesized that SMCR8 could reciprocally affect the stability of C9ORF72. If so, an *Smcr8* knockout mouse model could give more definitive insight into the in vivo functions of the C9orf72–Smcr8 protein complex. This strategy would circumvent the criticism that phenotypes seen in *C9orf72* mutant animals could be a result of disrupting *cis*-acting elements in the *C9orf72* genetic locus (Burberry et al. 2016; Joung et al. 2017b). To address the in vivo effects of Smcr8 loss, we applied CRISPR/Cas9-mediated genome editing in mouse zygotes. The murine *Smcr8* gene is composed of two exons encoding two different protein isoforms that are 935 and 785 amino acids in length, respectively. The first exon of *Smcr8* is common in both variants, encoding 84% of the longer protein isoform and encompassing the entire shorter protein isoform. We designed, validated, and selected two guide RNAs immediately downstream from the start codon of *Smcr8* (5′ targeting site) as well as two additional guide RNAs near the end of the first exon (3′ targeting site) (Fig. 2A). The on-target effect of the guide RNA cocktail would give rise to two distinct types of mutations: insertion/deletion (“indel”) mutations at the CRISPR\Cas9 target sites or a complete deletion of a 2200-nucleotide (nt) intervening sequence (“2-kb deletion”) between the 5′ and 3′ targeting sites (Fig. 2A).

After pronuclear injection of the guide RNA and *Cas9* mRNA, 32 mice were born and reached adulthood (*Smcr8* CRISPR F0 mice). We applied PCR and next-generation sequencing to genotype these animals, as illustrated in Figure 2A. To summarize, among the 32 mice, 15 (47%) carried both planned mutations, eight (25%) predominantly carried the 2-kb deletion, and one (3%) predominantly carried a 5′ indel mutation (Fig. 2B–D). To confirm the loss of *Smc8* in these mosaic founder animals, we designed three pairs of qPCR probes amplifying the 5′ CRISPR targeted site (*Smcr8_*a), the excised region located in between the two CRISPR sites (*Smcr8_*b), and a downstream untargeted region in exon 2 (*Smcr8_*c, illustrated in Fig. 2A), respectively. *Smcr8* transcripts containing an intact 5′ CRISPR target site were abolished in the peripheral blood of all founder mice, indicating the highly efficient on-target effect of the CRISPR–Cas9 nucleases. Transcripts normally located between the two CRISPR sites were detected in a subset of mice, indicating mosaicism in these animals. Transcript sequences downstream from the CRISPR target sites were detected but were significantly reduced in abundance, suggestive of nonsense-mediated decay of mutant transcripts (Fig. 2E, *P* < 0.0001 [****] by two-way analysis of variance [ANOVA] with Dunnett's multiple comparisons).

### *Smcr8* CRISPR F0 mice developed enlarged cervical lymph nodes, splenomegaly, and autoimmunity

**Figure 3.**
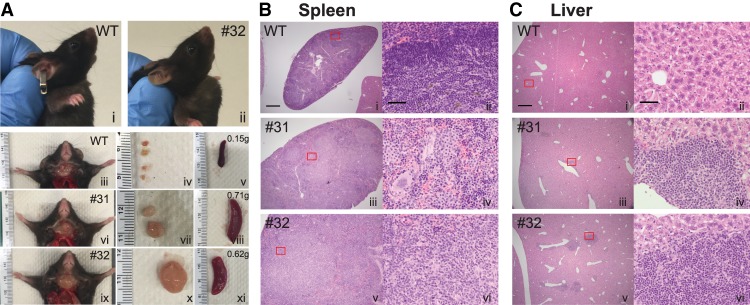
*Smcr8* CRISPR F0 mice develop enlarged cervical lymph nodes and immune cell infiltration in the spleen and liver. (*A*) Pictures of the cervical regions of an age-matched wild-type (WT) mouse (panel *i*) and the *Smcr8* CRISPR F0 mouse (mouse no. 32; panel *ii*) at 300 d old showing a cervical mass in the mutant mouse. Two of the *Smcr8* CRISPR F0 mice examined developed enlarged cervical lymph nodes and enlarged spleens. (Panels *iii–v*) Age-matched wild-type control mouse. (Panels *vi–viii*) *Smcr8* CRISPR F0 mouse number 31. (Panels *ix–xi*) F0 mouse number 32. (*B*,*C*) H&E staining of the spleens (*B*) and livers (*C*) from the same wild-type mouse and two *Smcr8* CRISPR F0 mice as shown in *A*. Bars: *B* (panel *i*), *C* (panel *i*), 500 µm; *B* (panel *ii*), *C* (panel *ii*), 50 µm.

Each of the *Smcr8* CRISPR F0 mice survived to adulthood. By 290 d of age, one of the 32 mice developed an externally visible cervical mass (Fig. 3A, panels i,ii), which was similar to those observed in a subset of *C9orf72*^−/−^ mice. We examined this particular mouse (mouse ID no. 32) together with one littermate *Smcr8* CRISPR F0 mouse (mouse ID no. 31) and one age-matched wild-type C57BL/6 mouse (wild type). Both of the *Smcr8* CRISPR F0 mice examined had enlarged cervical lymph nodes and enlarged spleens (0.15 g for the spleen in the wild type age-matched control and 0.71 g and 0.62 g, respectively, for the two *Smcr8* CRISPR F0 mice) (Fig. 3A, panels iii–xi). These phenotypes have been observed in additional *Smcr8* CRISPR F0 mice examined (Supplemental Fig. 3A). Subsequent histological studies showed that, while the white and red pulp boundary was clearly distinguishable in the wild-type control spleen, it was disrupted in both of the *Smcr8* CRISPR F0 mice examined (Fig. 3B). In addition, we also noted immune cell infiltration into the livers (Fig. 3C) of both mutant mice and into the lungs of one of the mutant mice (Supplemental Fig. 3B).

**Figure 4.**
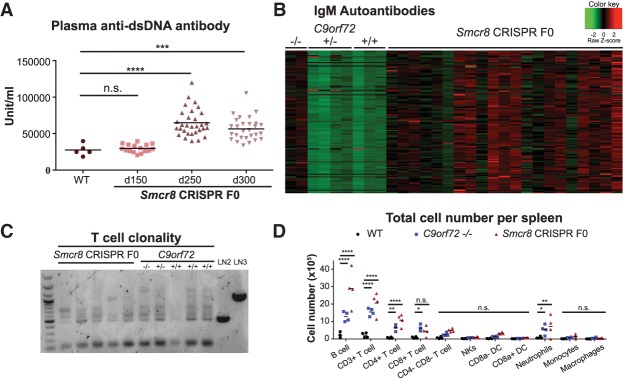
*Smcr8* CRISPR F0 mice developed signs of autoimmunity. (*A*) Anti-dsDNA (dsDNA) autoantibody concentration in the plasma of wild-type control or *Smcr8* CRISPR F0 mice at 150, 200, and 250 d old. (*B*) Heat map of plasma IgM activity against 124 different autoantigens in age-matched *C9orf72*^+/+^ (*n* = 3), *C9orf72*^+/−^ (*n* = 4), *C9orf72*^−/−^ (*n* = 2), and *Smcr8* CRISPR F0 (*n* = 15) mice. (*C*) T-cell clonality in the spleens of *Smcr8* CRISPR F0 mice and *C9orf72*^+/+^, *C9orf72*^+/−^, and *C9orf72*^−/−^ mice by PCR amplification of the somatic T-cell receptor β (TCRβ) locus. LN2 and LN3 represent ES cell lines derived from lymph node T cells harboring monoclonal TCRβ. (*D*) Flow cytometry analysis of total cell number of each of the indicated cell populations in the spleens of age-matched wild-type control, *C9orf72*^−/−^, and *Smcr8* CRISPR F0 mice. *n* = 4. (n.s.) Not significant; (*) *P* < 0.05; (**) *P* < 0.01; (***) *P* < 0.001; (****) *P* < 0.0001 by two-way ANOVA with Dunnett's multiple comparisons.

The phenotypes that we found in F0 mice could be explained by autoimmunity or lymphoma. As it has been shown that *C9orf72*^−/−^ mice developed autoimmunity, we first looked for signs of autoimmunity in the *Smcr8* mutant mice. To test this idea, we collected sequential plasma samples when the animals were 150, 250, and 300 d old and measured the level of anti-dsDNA autoantibody, a marker for autoimmunity (Burberry et al. 2016). At 150 d of age, there was no significant difference between the wild-type control (*n* = 5) and *Smcr8* CRISPR F0 (*n* = 16) mice. However, the anti-dsDNA antibody concentration gradually increased as the mutant mice aged, and, by 250 d of age, mutants displayed significantly more plasma anti-dsDNA antibodies. This phenotype persisted when tested 50 d later (Fig. 4A, *P* < 0.001 [***] and *P* < 0.0001 [****] by two-way ANOVA with Dunnett's multiple comparisons).

To further confirm that the elevated anti-dsDNA antibodies seen in the *Smcr8*^−/−^ mice were due to autoimmunity instead of dramatic synchronized cell death, we collected plasma from 250-d-old wild-type (*n* = 3), *C9orf72*^−/−^ (*n* = 2), and *Smcr8* CRISPR F0 (*n* = 21) mice and analyzed the levels of autoantibodies against a panel of 124 autoantigens (Li et al. 2005). Thirty-two of the IgM autoantibodies were found significantly elevated in *Smcr8* CRISPR F0 mouse plasma relative to wild-type mouse plasma (Fig. 4B; Supplemental Table 4). The IgM autoantibody activity in the plasma of 18 out of 21 *Smcr8* CRISPR F0 mice clustered more closely with the *C9orf72*^−/−^ samples (Fig. 4B). These observations were most consistent with the notion that *Smcr8* CRISPR F0 mice were developing an autoimmune condition.

To test an alternative hypothesis that animals were developing lymphoma, we examined T-cell clonality by analyzing V(D)J recombination (Whitehurst et al. 1999) in a subset of the CRISPR F0 mice (Fig. 4C). Although we noticed expansion of certain clones of T cells in three mutant mice, the T cells of each of the mice tested remained polyclonal. Furthermore, cell population analysis on the spleens revealed that, similar to *C9orf72*^−/−^ mice, the *Smcr8* CRISPR F0 mice had an increased total number of cells from multiple lineages, including CD19^+^ B cells, CD3^+^\CD4^+^ T cells, Cd11c^+^\CD8^−^ dendritic cells, and Ly6G^+^ neutrophils (Fig. 4D, not significant [n.s.], *P* < 0.05 [*], *P* < 0.01 [**], and *P* < 0.0001 [****] by two-way ANOVA with Dunnett's multiple comparisons). Our results indicated that the splenic cellular expansion in *Smcr8* mutant mice was polyclonal in nature, which was not consistent with lymphoma. Therefore, we concluded that, similar to previous observations in *C9orf72* mutant animals, autoimmunity was the most probable underlying cause of enlarged lymph nodes and splenomegaly seen in the *Smcr8* CRISPR F0 mice.

### Smcr8 reciprocally stabilizes the C9orf72 protein

**Figure 5.**
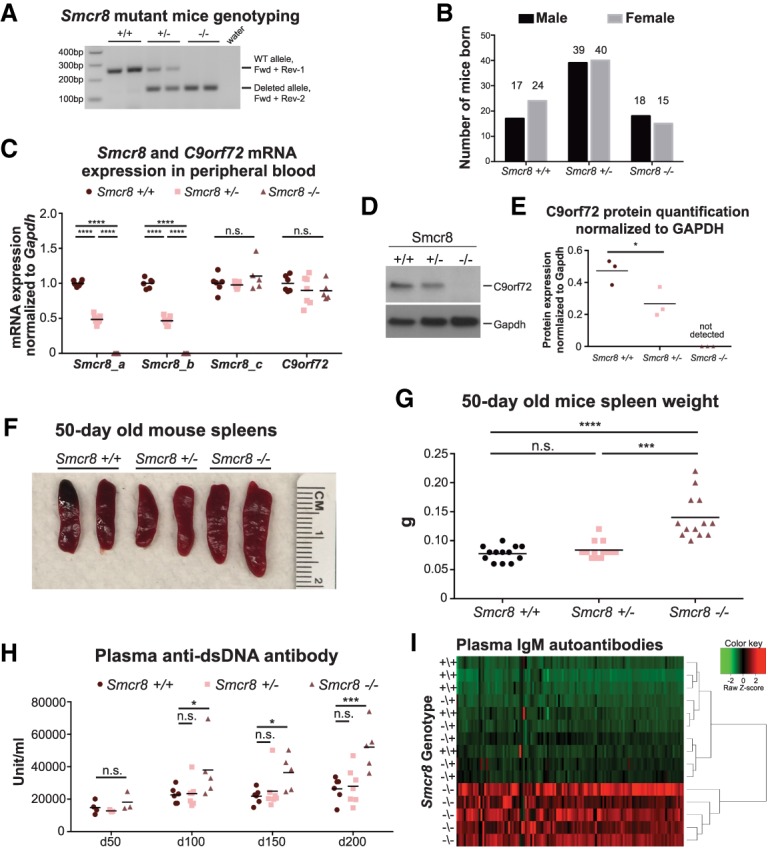
The splenomegaly and autoimmunity phenotypes are reproducibly caused by loss of Smcr8. (*A*) Examples of genotyping results. The upper band of 250 base pairs (bp) was amplified from the wild-type allele with probe sets Fwd and Rev-1, and the lower band of 150 bp was amplified from the 2-kb deleted allele with probe sets Fwd and Rev-2, as illustrated in Figure 2A. (*B*) The number of mice born of each genotype from *Smcr8*^+/−^ × *Smcr8*^+/−^ crosses. (*C*) *Smcr8* and *C9orf72* mRNA expression in the peripheral blood of 150-d-old mice. Three sets of probes amplifying three different regions of the *Smcr8* transcript, as illustrated in Figure 2A, were used. *n* = 6 *Smcr8*^+/+^; *n* = 7 *Smcr8*^+/−^; *n* = 5 *Smcr8*^−/−^. (n.s.) Not significant; (****) *P* < 0.0001 by two-way ANOVA with Dunnett's multiple comparisons. (*D*) Representative Western blot images for C9orf72 and Gapdh, respectively, in *Smcr8*^+/+^, *Smcr8*^+/−^, and *Smcr8*^−/−^ BMDMs. (*E*) Quantification of C9orf72 protein expression normalized to Gapdh in *Smcr8*^+/+^, *Smcr8*^+/−^, and *Smcr8*^−/−^ BMDMs. *n* = 3 per genotype. (*) *P* < 0.05 by two-tailed Student's *t*-test. (*F*) Representative images of 50-d-old mouse spleens from all three genotypes, as labeled. (*G*) The weights of freshly isolated spleens from 50-d-old mice. *n* = 13 for each genotype. (n.s.) Not significant; (***) *P* < 0.001; (****) *P* < 0.0001 by two-tailed Student's *t*-test. (*H*) Time-course assessment of plasma anti-dsDNA autoantibody concentration by ELISA. *n* = 6 *Smcr8*^+/+^; *n* = 7 *Smcr8*^+/−^; *n* = 5 *Smcr8*^−/−^. (n.s.) Not significant; (*) *P* < 0.05; (***) *P* < 0.001 by two-way ANOVA with Dunnett's multiple comparisons. (*I*) Heat map of plasma IgM activity against 124 different autoantigens in mice with different genotypes, as labeled. *n* = 5 for each genotype. Unsupervised clustering is shown at the *right*.

Given that C9ORF72 stabilizes SMCR8 and that *Smcr8* mutant mice developed phenotypes characteristic of the *C9orf72*^−/−^ animals, it was natural to ask whether Smcr8 reciprocally stabilizes the C9orf72 protein. We therefore set out to generate an *Smcr8* mutant mouse colony composed of mice with a single loss-of-function allele. We selected an *Smcr8* CRISPR F0 mouse harboring a 2-kb deletion allele for further studies. This particular allele corresponds to a 2276-nt deletion starting from the 15th nucleotide downstream from the ATG start codon, resulting in the deletion of 758 amino acids and frameshift of the remaining coding sequence. In this particular allele, 81% of the coding sequence for the longer protein isoform (or 97% for the shorter isoform) is completely lost without insertion of random nucleotide sequence. We bred the *Smcr8* CRISPR F0 C57BL/6 founder mouse containing this allele to wild-type C57BL/6 mice and intercrossed the subsequent *Smcr8*^+/−^ mice to obtain the second filial generation (F2) with three possible genotypes: wild type (+/+), heterozygous deletion (+/−), and homozygous deletion (−/−). The F2 mice were genotyped using a three-primer PCR strategy (illustrated in Figs. 2A, 5A). Mice of the three genotypes were born at the expected Mendelian ratios (Fig. 5B), further supporting the view that loss of *Smcr8* is not embryonic or early postnatal lethal.

Using the qPCR assays described above (Fig. 2A), we found a 50% reduction of *Smcr8* transcripts in *Smcr8*^+/−^ peripheral blood and an absence of detectable *Smcr8* transcript in the *Smcr8*^−/−^ samples (Fig. 5C, not significant [n.s.] and *P* < 0.0001 [****] by two-way ANOVA with Dunnett's multiple comparisons). Transcripts originating from exon 2 (downstream from the deleted region) were detected at comparable levels between the three genotypes, suggesting that this particular mutation did not result in nonsense-mediated decay (Fig. 5C). It is worth taking note that this particular 2-kb deletion allele in *Smcr8* did not lead to a detectable effect on *C9orf72* mRNA expression (Fig. 5C).

To ask whether this mutation in Smcr8 affected C9orf72 protein levels, we generated BMDMs from *Smcr8*^+/+^, *Smcr8*^+/−^, and *Smcr8*^−/−^ animals (*n* = 3 per genotype) and measured C9orf72 protein expression by Western blot. We found a 50% reduction of C9orf72 protein in *Smcr8*^+/−^ BMDMs and a complete loss of C9orf72 in *Smcr8*^−/−^ BMDMs (Fig. 5D,E, *P* < 0.05 [*] by two-tailed Student's *t*-test). Therefore, we concluded that the protein stabilities of C9orf72 and Smcr8 are reciprocally dependent on each other.

### Splenomegaly and autoimmunity reproducibly result from loss of Smcr8

The genetic locus encoding *C9orf72* is highly conserved between humans and mice and is in close proximity to a long noncoding RNA (lncRNA) locus (*EMICERI*) and the gene encoding interferon κ (*Ifnk*) (Joung et al. 2017b). It had been demonstrated recently that expression of lncRNAs found in the *EMICERI* locus are capable of affecting the expression of nearby genes, including *C9orf72* and *Ifnk* (Joung et al. 2017b). Therefore, one outstanding concern surrounding established *C9orf72* loss-of-function mutant mouse models is that the immune phenotypes associated with these mice could be the result of altering the expression of *cis*-acting lncRNAs and genes found near the *C9orf72* locus. As C9orf72 and Smcr8 proteins stabilize each other, we reasoned that by studying the phenotype of *Smcr8* mutant animals harboring this 2-kb deletion mutation, we could address the in vivo function of the C9orf72–Smcr8 protein complex without interrupting the local architecture of the *C9orf72* genetic locus and therefore avoid the confounding factors mentioned above.

We noted that by 50 d of age, *Smcr8*^−/−^ mice had developed significantly enlarged spleens (Fig. 5F,G, not significant [n.s.], *P* < 0.001 [***], and *P* < 0.0001 [****] by two-tailed Student's *t*-test). Histological study showed that *Smcr8*^−/−^ F2 animals reproducibly displayed disrupted spleen structure (Supplemental Fig. 3C) and lymphocyte infiltration into the liver (Supplemental Fig. 3D) and lungs (Supplemental Fig. 3E). By 100 d of age, *Smcr8*^−/−^ F2 animals developed signs of autoimmunity, with significantly more elevated plasma anti-dsDNA antibody concentration compared with their littermate *Smcr8*^+/+^ controls, a phenotype that persisted until at least 200 d of age (Fig. 5H, not significant [n.s.], *P* < 0.05 [*], and *P* < 0.001 [***] by two-way ANOVA with Dunnett's multiple comparisons). We next subjected the plasma from 250-d-old mice to autoantibody array analysis (*n* = 5 for each genotype). Sixty-three of 124 IgM autoantibodies were found to be significantly elevated in *Smcr8*^−/−^ plasma relative to *Smcr8*^+/+^ plasma, eight of which were also significantly elevated in *Smcr8*^+/−^ plasma (Supplemental Table 5, two-way ANOVA with Dunnett multiple comparison). Moreover, *Smcr8*^−/−^ samples formed a distinct group apart from the *Smcr8*^+/+^ and *Smcr8*^+/−^ samples by hierarchical clustering of the autoantibody quantification (Fig. 5I).

In addition, we also observed a modest but significant increase in the abundance of the autophagy marker protein p62 in *Smcr8*^−/−^ spleen samples compared with controls in two independent cohorts of mice (Supplemental Fig. 5C,D), a phenotype that has been reported previously in C9orf72 loss-of-function animals (O'Rourke et al. 2016). In these regards, the phenotypes observed in *Smcr8*^−/−^ animals were indistinguishable from those routinely found in *C9orf72*^−/−^ mice (Burberry et al. 2016). While we did also detect significant elevation in autoimmune IgG titers in *Smcr8* mutant mice (Supplemental Figs. 4 [for *Smcr8* CRISPR F0 mice], 5A [for F2 mice]; Supplemental Tables 6, 7), it was not as severe as that observed in the *C9orf72*^−/−^ animals. In addition, contrary to the *C9orf72*^−/−^ animals, we did not observe a significant induction of inflammatory cytokine production in *Smcr8*^−/−^ mice (Supplemental Fig. 5B), leaving open the question of whether this important and reproducible phenotype in *C9orf72* mutant animals is due to a subtle distinction in genetic background or *cis*-acting effects of the *C9orf72* mutation.

### *Smcr8*^−/−^ mice develop anemia and neutrophilia

It had been shown previously that the *C9orf72*^−/−^ mice developed anemia, thrombocytopenia, and neutrophilia (Burberry et al. 2016). To see whether these phenotypes were reproduced in *Smcr8*^−/−^ mice, we performed sequential complete blood cell counts on the peripheral blood of *Smcr8*^+/+^ (*n* = 6), *Smcr8*^+/−^ (*n* = 7), and *Smcr8*^−/−^ (*n* = 5) mice. The same cohort of mice was repeatedly bled every 50 d until 250 d of age. Although total red blood cell counts were not significantly different between the genotypes across the time points examined (Supplemental Fig. 6A), multiple signs of anemia did develop in the *Smcr8*^−/−^ mice at 150 d of age and persisted until at least 250 d of age, which included lower concentrations of hemoglobin, a lower hematocrit, a lower mean corpuscular volume, lower mean corpuscular hemoglobin, and higher red blood cell width distribution. In total, these observations are consistent with *Smcr8*^−/−^ animals developing a microcytic anemia similar to that found in *C9orf72* mutant animals (Supplemental Fig. 6B–F). The platelet counts in the *Smcr8*^−/−^ mice also became significantly reduced by 250 d of age (Supplemental Fig. 6G). At this age, the mutant mice also developed higher levels of total white blood cells in their peripheral blood (Supplemental Fig. 6H), which was associated with a decreased percentage of lymphocytes (Supplemental Fig. 6I,J) and an increased total number (Supplemental Fig. 6K) and percentage (Supplemental Fig. 6L) of neutrophils. Therefore, loss of *Smcr8* led to changes in peripheral blood cell composition that were indistinguishable from those observed in the *C9orf72*^−/−^ mice (Burberry et al. 2016), including anemia, thrombocytopenia, and neutrophilia.

### Loss of Smcr8 enhanced lysosomal exocytosis in macrophages

**Figure 6.**
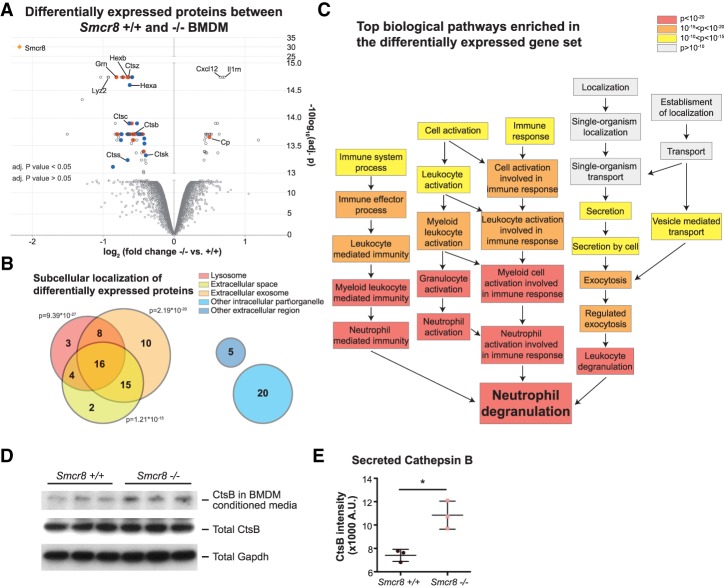
Loss of Smcr8 results in down-regulation of lysosomal lumen protein expression. (*A*) Differentially expressed proteins in *Smcr8*^−/−^ BMDMs compared with wild-type BMDMs by quantitative mass spectrometry. Data are represented as log_2_ of protein expression fold change in *Smcr8*^−/−^ versus *Smcr8*^+/+^ on the *X*-axis and log_10_ of adjusted *P*-value by moderated *t*-test on the *Y*-axis. (Orange diamond) Smcr8; (blue dots) lysosome-located genes; (red dots) proteins that can be found in lysosomes, extracellular space, and extracellular exosomes. (*B*) Summary of gene ontology cellular component enrichment analysis of differentially expressed proteins in *Smcr8*^−/−^ BMDMs. (*C*) Top biological processes that are enriched in the list of differentially expressed proteins in *Smcr8*^−/−^ BMDMs. (*D*) Secreted cathepsin B (CtsB) in conditioned medium, total CtsB, and total Gapdh as a loading control in *Smcr8*^+/+^ and *Smcr8*^−/−^ BMDMs. (*E*) Quantification of secreted CtsB levels in the BMDM conditioned medium. (*) *P* < 0.05 by two-tailed Student's *t*-test.

The cell biological functions of the C9orf72–Smcr8 protein complex have remained controversial. In particular, how this complex functions to suppress chronic inflammation and autoimmunity remains unknown. To generate further hypotheses, we took an unbiased approach and looked for differentially expressed proteins in *Smcr8*^−/−^ BMDMs relative to *Smcr8*^+/+^ controls by quantitative mass spectrometry. We selected macrophages as our cellular model for the following reasons: First, complete blood cell counts (Supplemental Fig. 6) suggested that loss of Smcr8 resulted in abnormalities in myeloid cells, including macrophages. Second, it had been reported previously that *C9orf72*^−/−^ macrophages and microglia (resident macrophages in the central nervous system) showed changes in lysosomal function (O'Rourke et al. 2016). We generated independent BMDM cultures from three distinct mice of each genotype. Protein samples were isolated from mature BMDMs after 8 d of differentiation in medium supplemented with macrophage colony stimulation factor (M-CSFs), and the whole proteome was analyzed by TMT quantitative mass spectrometry. A total of 8096 proteins was identified with at least two distinct peptide sequences (Supplemental Table 8). A dramatic loss of peptides originating from Smcr8 was seen in the *Smcr8*^−/−^ samples (Fig. 6A; Supplemental Table 9), confirming the depletion of Smcr8 in our mouse model. Eighty-nine additional proteins were found differentially expressed between *Smcr8*^−/−^ and *Smcr8*^+/+^ BMDMs (defined as adjusted *P*-value of <0.05 by moderated *t*-test) (Supplemental Table 9).

We queried the list of significantly differentially expressed gene products with the online gene ontology enrichment analysis tool (http://geneontology.org/page/go-enrichment-analysis) and looked for their subcellular localization (cellular component). This list of differentially expressed gene products was highly enriched for proteins located to the lysosome (31 out of 90; *P* = 9.39 × 10^−27^), the extracellular space (37 out of 90; *P* = 1.21 × 10^−15^), and extracellular exosomes (49 out of 90; *P* = 2.19 × 10^−20^) (Fig. 6B). Among the 31 lysosome-located gene products, 30 were found decreased with loss of Smcr8. Among the 10 lysosomal hydrolases cathepsins that were detected in this experiment, five were significantly decreased in *Smcr8*^−/−^ BMDMs. The remaining differentially expressed proteins were either found in other intracellular regions or organelles (20 proteins), found in other extracellular regions (five proteins), or not annotated (seven proteins). Next, we asked which biological processes were likely to be disrupted in the *Smcr8*^−/−^ macrophages using the same informatics tool. Differentially expressed proteins were significantly enriched for murine ganglioside and oligosaccharide catabolic processes (four genes; *P* = 1.85 × 10^−4^). As we noticed that the human genes were better annotated for certain pathways, we looked for human homologs of all of the significantly changed genes in our mass spectrometry experiment. This “humanized” list was highly enriched for human immune cell activation pathways (30 out of 83 mapped genes), converging to the neutrophil degranulation pathway (*P* = 4.92 × 10^−22^) (Fig. 6C). The genes implicated in these immune activation pathways fully overlapped with the lysosomal-located genes, consistent with the importance of the lysosome in leukocyte degranulation and immune activation (Alter et al. 2004; Betts and Koup 2004). Unfortunately, peptides originating from C9orf72 were not detected in any genotype of the sample analyzed in this particular experiment, rendering it impossible to independently confirm in this unbiased context whether reduced Smcr8 levels led to reduced C9orf72 levels.

Next, we asked whether the reduction of total cellular expression of lysosomal proteins could be due to enhanced lysosomal exocytosis. To begin addressing this question, we looked at the secretion of cathepsin B (CtsB), a lysosomal thiol protease, by measuring its concentration in BMDM culture medium. We produced three independent BMDM differentiations from *Smcr8*^+/+^ and *Smcr8*^−/−^ mice for this purpose. After 7 d of differentiation, 50,000 cells were replated in a well of a 96-well plate. The day following replating, cells were washed with PBS and refed with fresh BMDM medium. Cell culture supernatant samples were collected 1 d later and analyzed by Western blot. We found that the amount of extracellular CtsB was significantly increased in the cell culture supernatant of *Smcr8*^−/−^ BMDMs (Fig. 6D,E, *P* < 0.05 [*] by two-tailed Student's *t*-test) compared with controls.

**Figure 7.**
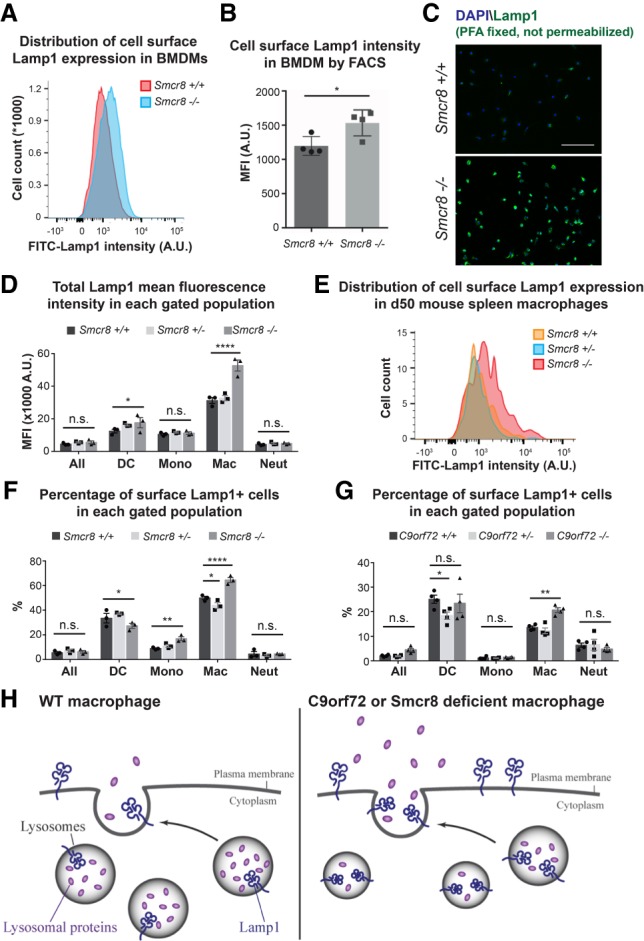
Loss of Smcr8 results in enhanced lysosomal exocytosis marked by increased cell surface Lamp1 expression. (*A*,*B*) Cell surface Lamp1 expression by flow cytometry from BMDMs derived from *Smcr8*^+/+^ and *Smcr8*^−/−^ mice. *n* = 4 for each genotype. (*A*) Representative histogram of FITC-Lamp1 intensity on the surface of *Smcr8*^+/+^ (red) and *Smcr8*^−/−^ (blue) BMDMs. (*B*) Quantification of cell surface FITC-Lamp1 intensity on BMDMs differentiated from four different mice for each genotype. (*) *P* < 0.05 by two-tailed Student's *t*-test. (*C*) Representative image of Lamp1 immunofluorescent staining on PFA-fixed but not permeabilized BMDMs from *Smcr8*^+/+^ and *Smcr8*^−/−^ mice. (*D*) Total Lamp1 expression in each of the indicated splenocyte populations in 50-d-old *Smcr8*^+/+^, *Smcr8*^+/−^, and *Smcr8*^−/−^ presymptomatic mice analyzed by flow cytometry. *n* = 3 for each genotype. (*E*) Representative histogram of cell surface Lamp1 expression intensity in the same set of mice as in *D*. (*F*) The percentage of surface Lamp1-positive cells in each gated cell population from the spleens of the same set of *Smcr8* cohort of mice as in *D*. (*G*) The percentage of cell surface Lamp1-positive cells in each gated population from the spleens of 50-d-old *C9orf72*^+/+^, *C9orf72*^+/−^, and *C9orf72*^−/−^ mice. *n* = 4 for each genotype. (n.s.) Not significant; (*) *P* < 0.05; (**) *P* < 0.01; (***) *P* < 0.001 by two-way ANOVA with Dunnett's multiple comparisons. (*H*) Proposed model for the cell biological function of the C9orf72–Smcr8 protein complex. Under normal conditions, intracellular lysosomes were labeled by lysosome membrane-associated protein Lamp1 and contained a certain amount of lysosomal luminal hydrolases, among which CtsB is one of the components. Some lysosomes undergo exocytosis, which results in the secretion of lysosomal luminal proteins and exposure of the luminal epitope of Lamp1 on the cell surface. While the C9orf72–Smcr8 protein complex function is compromised, lysosomal exocytosis is intensified as marked by (1) decreased total intracellular lysosomal protein content, (2) enhanced secretion of lysosomal luminal protein, and (3) the increased presence of the luminal epitope of Lamp1 exposed on the cell surface. Our model suggests that the C9orf72–Smcr8 protein complex could play a negative regulatory role in lysosomal exocytosis.

Cells from the hematopoietic lineage are among the few cell types that use modified lysosomes as their secretory organelles, which are important in modulating the immune responses (Blott and Griffiths 2002). In cytotoxic T cells and natural killer cells, the cytotoxic granules are essentially secretory lysosomes (Alter et al. 2004; Betts and Koup 2004). Cell surface expression of Lamp1 (also known as CD107a) has been widely used as a marker for lysosomal exocytosis (Alter et al. 2004; Betts and Koup 2004). We therefore investigated expression of lamp1 on the surface of *Smcr8*^+/+^ and *Smcr8*^−/−^ BMDMs. Independent BMDM cultures were generated from four mice of each genotype. Mature BMDMs were washed with ice-cold PBS to suppress endocytosis. To specifically label cell surface Lamp1 that resulted from exocytosis, we stained live cells with an antibody specifically recognizing the portion of the protein found in the lysosomal lumens. When analyzed by flow cytometry, we found that BMDMs derived from *Smcr8*^−/−^ mice expressed significantly more of this Lamp1 antigen on the cell surface when compared with *Smcr8*^+/+^ cells (Fig. 7A,B, *P* < 0.05 [*] by two-tailed Student's *t*-test). We also performed immunofluorescent staining of fixed but unpermeabilized BMDMs using Lamp1 antibody recognizing the same luminal epitope. While the Lamp1 signal was barely detected on *Smcr8*^+/+^ BMDMs, we detected a strong signal on unpermeabilized *Smcr8*^−/−^ BMDMs (Fig. 7C).

We next proceeded to ask whether the increase in surface Lamp1 expression that we observed in BMDMs in vitro also occurred in vivo within *Smcr8*^−/−^ animals. To this end, we collected and dissociated spleens from 50-d-old presymptomatic mice and examined Lamp1 expression in different myeloid lineages of cells by flow cytometry. When splenocytes were fixed and permeabilized prior to antibody labeling, we observed that *Smcr8*^−/−^ F4-80^+^ macrophages as well as dendritic cells expressed more total Lamp1 protein (Fig. 7D, not significant [n.s.], *P* < 0.05 [*], and *P* < 0.0001 [****] by two-way ANOVA with Dunnett's multiple comparisons). When unpremeabilized splenocytes were assessed, we also found that a significantly larger proportion of *Smcr8*^−/−^ macrophages expressed Lamp1 on their cell surface (Fig. 7E,F, not significant [n.s], *P* < 0.05 [*], *P* < 0.01 [**], and *P* < 0.0001 [****] by two-way ANOVA with Dunnett's multiple comparisons) compared with cells from their wild-type control littermates. These findings support the hypothesis that Smcr8 also restricts the lysosomal exocytosis pathway in splenic macrophage populations in vivo.

As our results suggested convergent roles for C9orf72 and Smcr8, we asked whether the enhanced accumulation of lysosomal components on the cell surface that we found in *Smcr8*^−/−^ splenic macrophages could also be observed in *C9orf72*^−/−^ splenic macrophages. Upon examination of cell surface Lamp1 by flow cytometry, we found that a significantly higher proportion of day 50 *C9orf72*^−/−^ splenic macrophages displayed cell surface Lamp1 expression compared with *C9orf72*^+/+^ and *C9orf72*^+/−^ macrophages (Fig. 7G, not significant [n.s.], *P* < 0.05 [*], and *P* < 0.01 [**] by two-way ANOVA with Dunnett's multiple comparisons).

## Discussion

As *C9ORF72* insufficiency may be a contributor to ALS in patients harboring the repeat expansion at this locus, understanding the cell biological function of the C9ORF72 protein is of critical importance. Our findings confirm observations by others that *C9ORF72* reproducibly interacts with the, until recently, largely unstudied proteins SMCR8 and WDR41. We also extend the knowledge surrounding this complex by demonstrating that these interactions are specific to C9-long and occur in a variety of cell types, including human pluripotent stem cells and motor neurons. A number of groups, including our own (Sullivan et al. 2016; Webster et al. 2016; Yang et al. 2016; Jung et al. 2017), have found that these three interacting proteins further interact with the autophagy initiation complex composed of FIP200–ULK1–ATG13, leading to the proposal that C9ORF72 somehow functions in the autolysosomal pathway (O'Rourke et al. 2016; Sullivan et al. 2016; Webster et al. 2016; Yang et al. 2016; Jung et al. 2017). While multiple hypotheses have been put forward concerning which cell biological aspects of this pathway are regulated by C9ORF72, little consensus has been reached.

In contrast, relatively more agreement has emerged concerning the organismal effects of reduced C9ORF72 function. We, along with several other groups, have examined mice harboring loss-of-function variants in the *C9ORF72* ortholog and found that these animals developed autoimmune and inflammatory diseases of varying severity. The remaining criticism of these collective studies has been that, in addition to disrupting expression of *C9orf72*, the alleles studied thus far also might have disrupted expression of a lncRNA that coregulates physically linked genes known to function in the immune system (Joung et al. 2017a). Our finding that *C9orf72* and *Smcr8* mutually stabilize one another provided a key opportunity to resolve whether removing the function of this complex was indeed sufficient to lead to inflammatory and autoimmune disease. By generating mice with loss-of-function mutations in *Smcr8*, we could deplete mice of the C9orf72 protein while leaving the physical *C9orf72* gene and transcript intact and then ask whether these animals developed a similar autoimmune and inflammatory disease. We found that both F0 mosaic founders and subsequent F2 *Smcr8*^−/−^ animals developed autoimmune phenotypes that mimicked the *C9orf72* loss-of-function mutants on a similar genetic background. While gene-editing approaches can have off-target effects, our findings would seem to resolve any lingering doubt concerning whether C9orf72 and its molecular collaborator, Smcr8, normally function to suppress autoimmunity. First and foremost, our findings further our previous conclusion (Burberry et al. 2016) that any therapeutic efforts to reduce the abundance of the gain-of-function products from patients carrying the *C9ORF72* repeat expansion should not come at the expense of reducing expression of its normal gene products.

Confirmation that the main function of the C9orf72–Smcr8 protein complex is to regulate immune system function further raises the possibility that C9ORF72 insufficiency caused by the presence of the repeat expansion mutation could alter an individual's immune system function. In fact, it has been proposed that autoimmune and inflammatory biology is a key disease mechanism in ALS (Pagani et al. 2011). It has been observed that immunoglobulins purified from the sera of some ALS patients were neurotoxic (Pullen et al. 2004; Demestre et al. 2005). This toxicity mediated by ALS sera has been associated with elevated autoantibodies recognizing various nervous system self-antigens, including myelin (Lisak et al. 1975), synaptic proteins (Demestre et al. 2005; Pagani et al. 2006), voltage-gated calcium channels (Delbono et al. 1991; Smith et al. 1992; Kimura et al. 1994), neurofilament (Couratier et al. 1998), and vascular components (Greiner et al. 1992). More recently, retrospective studies analyzing patient records have shown that individuals with ALS\FTD, especially those carrying the *C9ORF72* repeat expansions, were significantly more likely to have been diagnosed previously with autoimmune abnormalities (Turner et al. 2013; Miller et al. 2016). Whether patients with the *C9ORF72* repeat expansion have changes to their immune system that could contribute to autoimmune phenoytpes observed previously in ALS patients warrants future investigations.

Based on our findings in vivo, we went on to use quantitative deep-scale proteomics to explore the function of Smcr8 in macrophages and found that they may negatively regulate lysosomal exocytosis. We found that loss of Smcr8 significantly reduced the abundance of a group of lysosomal lumen proteins inside macrophages, accompanied by an elevated presence of the lysosomal lumen protein CtsB in the extracellular space. In addition, the cell surface expression of luminal Lamp1, a broadly used marker for lysosomal exocytosis (Alter et al. 2004; Betts and Koup 2004), was found to be significantly increased in splenic macrophages acutely isolated from *Smcr8*^−/−^ and *C9orf72*^−/−^ mice. Consistent with the idea that the C9orf72–Smcr8 protein complex may function in the lysosome compartment, it had been proposed recently that the C9orf72 and Smcr8 protein complex may affect lysosomal homeostasis in *Caenorhabditis elegans* (Corrionero and Horvitz 2018). In addition, the third component of the C9orf72 protein complex, Wdr41, had been proposed to localize to the lysosome membrane (Schroder et al. 2007). Our finding that there are modestly increased levels of p62 in the spleens of both *Smcr8* and *C9orf72* mutant mice further supports the notion that these two gene products function in this pathway.

The identification of a role for the C9orf72–Smcr8 protein complex in lysosomal exocytosis is intriguing, as it could provide a link between the cell biological function of the protein complex and the in vivo autoimmune phenotypes seen in mutant animals. Lysosomal exocytosis is thought to exist only in certain cell types, mainly in the immune system (Blott and Griffiths 2002). This process is essential for T-cell and natural killer cell degranulation and cytotoxic activities (Betts et al. 2003; Alter et al. 2004) and plays a role in antigen presentation and cytokine secretion (Blott and Griffiths 2002; Luzio et al. 2007; Ge et al. 2015). Therefore, a disruption in normal lysosomal exocytosis may affect an individual's susceptibility to developing autoimmune and inflammatory diseases. Although this is not in the scope of our study, if the autoimmune phenotypes seen in the C9orf72–Smcr8 mutant animals could be rescued by suppressing lysosome exocytosis, it would strengthen a causative link between the altered cell biological function and in vivo phenotypes caused by the loss of the functional C9orf72–Smcr8 protein complex, a long-standing important question in the field. In the future, it will of interest to determine whether it is loss of *C9orf72* function in a particular lineage, such as macrophage or dendritic cells, that drives the inflammatory phenotypes that we observed.

In closing, we note that a recent ALS phase III clinical trial showed efficacy for a combination therapy containing masitinib and riluzole. Patients treated with this regimen had a significantly improved interim outcome with a slower rate of functional decline when assessed by the ALS functioning rate scale (ALSFRS) *R*score and an improved quality of life (Khalid et al. 2017). It is worth noting that masitinib is a selective inhibitor for the tyrosine kinase KIT and functions to inhibit mast cell degranulation (Dubreuil et al. 2009), a process dependent on lysosomal exocytosis, which we showed in this study to be negatively regulated by the C9orf72–Smcr8 protein complex.

## Materials and methods

### Gene targeting for making C9ORF72-HA transgenic hES cell lines

Zinc finger nuclease targeting the *AAVS1* locus was described previously (Hockemeyer et al. 2009; Lombardo et al. 2011). Donor targeting vector was modified from the donor vector used in the same study by inserting C9ORF72 cDNA with a C-terminal HA tag driven by the CAG promoter between the end of the PGK puromycin selection cassette and the 3′ homology arm. hES cells were dissociated to single cells with Accutase (Innovative Cell Technologies, AT-104-500). One microgram of ZFN plasmid and 5 µg of donor plasmid were transfected into 5 million dissociated cells by using the Neon transfection system from Invitrogen. Cells were plated on Matrigel-coated (Thermo Fisher Scientific, 08774552) 10-cm dishes in mTeSR1 (Stem Cell Technologies, 05850) supplemented with rock inhibitor (1 µM final concentration; DNSK, Y27632) on the day of transfection. Puromycin-resistant colonies were individually picked and PCR-analyzed for proper transgene insertion.

### cDNA expression vector and siRNA

SMCR8-MycDDK and SMCR8-GFP in pCMV6 backbones were from Origene (RG212285). The following siRNAs were obtained from Thermo Fisher Scientific: BLOCK-IT Alexa fluor red for control (no. 14750), siRNA targeting C9ORF72 (4392420-s47492), SMCR8 (4392420-s44397), and WDR41 (4392420-s30567).

HEK293 cells were cultured in DMEM\F12 with 10% fetal bovine serum. Cells were passaged the day before and aimed for 70%–80% confluency for the time of transfection. For overexpression vector transfection, 1 µg for each DNA construct was diluted in 200 µL of Opti-Mem I (Life Technologies, 11058021) and 6 µL of polyethylenimine at 1 mg/mL (Thermo Fisher Scientific, NC9197339) for one well of a six-well plate. An empty vector was added to balance the total amount of transfected DNA. For siRNA transfection, 100 pmol of siRNA was diluted in 245 µL of Opti-Mem, and 5 µL of RNAiMax (Life Technologies, 13778075) was diluted in 245 µL of Opti-Mem for one well of a six-well plate. The cocktail was incubated for 15 min at room temperature and then added to the cells. Samples were collected and analyzed 4 d after transfection.

### Immunoprecipitation

Cells were washed on the plate with PBS and lysed in Pierce immunoprecipitation lysis buffer (Thermo Fisher Scientific, PI87787) supplemented with Pierce Halt protease and phosphatase inhibitor cocktail (no. 78443). Immunoprecipitation was performed by incubating protein lysates with antibody-coupled magnetic beads (anti-HA magnetic beads [Cell Signaling Technology, 11846S] and anti-myc magnetic beads [Thermo Fisher Scientific, PI88842]) overnight at 4°C with rotation followed by three washes with lysis buffer. Samples were either brought for mass spectrometry analysis or eluted with SDS-PAGE gel loading buffer (Thermo Fisher Scientific, 50194470) for Western blot analysis; otherwise, cells were lysed in RIPA buffer supplemented with protease and phosphatase inhibitor cocktail for direct Western blot analysis.

### Smcr8 CRISPR guide design and validation and mouse generation

CRISPR guides were designed using the online CRISPR design tool (Hsu et al. 2013). Each selected guide sequence was inserted between the Sp6 transcriptase promoter and terminator. The guide was then transcribed into mRNA using MEGAscript Sp6 transcription kit (Ambion, AM1330M) and purified using MEGAclear transcription clean-up kit (Ambion, AM1908). Wild-type *Cas9* nuclease mRNA was obtained from System Biosciences, Inc. (CAS500A-1). The purified guide RNAs and *Cas9* mRNA were injected into mouse blastocysts at the Harvard Genome Modification Facility. The exact mutation was analyzed by MiSeq next-generation sequencing of PCR amplicons of the target site at the Broad Institute walk-up sequencing platform. The sequencing result was analyzed using the online tool OutKnocker 1.2 (http://www.outknocker.org).

### Mouse blood work and tissue processing

The complete blood count, cytokine measures, and anti-dsDNA antibody titration were performed as described previously (Burberry et al. 2016). Autoantibody reactivity against a panel of 124 autoantigens was measured using an autoantigen microarray platform developed by University of Texas Southwestern Medical Center (https://microarray.swmed.edu/products/category/protein-array) (Li et al. 2005, 2007). Mouse tissue collection, fixation, sectioning, and staining were done as described (Burberry et al. 2016).

### RNA extraction and qPCR

Total RNA extraction, generation of complementary DNA, and qPCR were performed as described previously (Burberry et al. 2016).

### Mouse bone marrow macrophage culture

Mouse bone marrow macrophages were made following an established protocol (Weischenfeldt and Porse 2008). Briefly, bone marrow was extracted from the femurs of both hind legs and cultured in RPMI-1640 containing 10% fetal bovine serum and glutamax and supplemented with M-CSFs (Peprotech) at 20 ng/mL.

### Fluorescence-activated cell sort (FACS)

Splenocyte isolation, antibody staining, gating, and analysis were performed as described previously (Burberry et al. 2016). The monoclonal antibody recognizing the luminal epitope of Lamp1 was obtained from Biolegend (clone 1D4B).

### Primary antibodies

The following primary antibodies were used: HA (Cell Signaling Technology, CST3724), Flag (SigmaAldrich, F1804), Smcr8 (Abcam, ab202283), Wdr41 (Thermo Fisher Scientific, PA524352), and Lamp1 (Santa Cruz Biotechnology, sc-19992).

### Mass spectrometry

Detailed mass spectrometry methods and data analysis are described in the Supplemental Material. Briefly, for protein interaction analyses, samples were reduced, alkylated, and digested on bead with trypsin or in solution for global proteomics analysis. Desalted peptides were labeled with TMT (10-plex or 6-plex) reagents (Thermo Fisher Scientific), fractionated using basic reversed-phase chromatography (global proteomics only), and analyzed by online nanoflow liquid chromatography-tandem mass spectrometry using a Q Exactive Plus mass spectrometer (Thermo Fisher Scientific) coupled online to a Proxeon Easy-nLC 1200 (Thermo Fisher Scientific). All data were analyzed using Spectrum Mill software package version 6.1 prerelease (Agilent Technologies). Data were filtered to consider only proteins with two or more unique peptides and median-normalized.

### Statistics

All statistical analyses were performed using GraphPad Prism. Two-tailed Student's *t*-test was applied for comparison of one single parameter between two groups. A moderated *t*-test was applied for all mass spectrometry analysis. Unless specified otherwise, one-way ANOVA was used for comparison of one single parameter between multiple groups, and two-way ANOVA with Dunnett's test was applied for comparison of two parameters between multiple groups. Tukey's multiple comparison test was applied to compare the mean of every other group, and Dunnett's multiple comparison test was applied to compare every mean with that of a control group.

## Supplementary Material

Supplemental Material
