# Supplementary material for: The C9orf72-interacting protein Smcr8 is a negative regulator of autoimmunity and lysosomal exocytosis
Source: Genes Dev. 2018 Jul 1;32(13-14):929–43. doi: 10.1101/gad.313932.118 (PMC6075033; doi:10.1101/gad.313932.118)
Supplement: Supplemental Material [file supp_32_13-14_929__index.html]

The C9orf72-interacting protein Smcr8 is a negative regulator of autoimmunity and lysosomal exocytosis — Supplemental Material 

# The C9orf72-interacting protein Smcr8 is a negative regulator of autoimmunity and lysosomal exocytosis

## Supplemental Material

- Supplemental\_Table\_S1.xlsx
- Supplemental\_Table\_S2.xlsx
- Supplemental\_Table\_S3.xlsx
- Supplemental\_Table\_S4.xlsx
- Supplemental\_Table\_S5.xlsx
- Supplemental\_Table\_S6.xlsx
- Supplemental\_Table\_S7.xlsx
- Supplemental\_Table\_S8.xlsx
- Supplemental\_Table\_S9.xlsx
- Supplemental\_figure\_legends.docx
- Supplemental\_Fig\_S1.ai
- Supplemental\_Fig\_S4.ai
- Supplemental\_Fig\_S2.ai
- Supplemental\_Fig\_S5.ai
- Supplemental\_Fig\_S3.tif
- Supplemental\_Fig\_S6.ai
