# Supplementary material for: The C9orf72-interacting protein Smcr8 is a negative regulator of autoimmunity and lysosomal exocytosis
Source: Genes Dev. 2018 Jul 1;32(13-14):929–43. doi: 10.1101/gad.313932.118 (PMC6075033; doi:10.1101/gad.313932.118)
Supplement: Supplemental Material [file supp_gad.313932.118_Supplemental_figure_legends.docx]

**Supplemental figure 1. Validation of C9ORF72-HA overexpression stem cell lines and western blot validation of C9ORF72 long isoform interacting proteins**

(A) Top: illustration of genotyping strategy used to validate proper *C9ORF72-HA* expression cassette in the *AAVS1* locus. Bottom: PCR validation using the indicated primers. *TRH* is a set of primer that amplifies an intact region for control of DNA quality. (B) qPCR validation of *C9ORF72* overexpression in the established cell lines. n=3 for each line. ***:p<0.001 by t-test. Error bar represents standard deviation. (C and D) Western blot validation of C9ORF72-HA expression before and after anti-HA immunoprecipitation of the indicated cell lines with anti-HA (C) and anti-C9ORF72 (D) antibodies. (E and F) Western blot validation of C9ORF72 interaction with SMCR8 (E) and WDR41 (F) in human embryonic stem cells. (G) C9ORF72 interacting proteins in human motor neurons. The control and C9ORF72-long-HA expressing cell lines were differentiated into motor neurons and then underwent anti-HA immunoprecipitation and quantitative mass spectrometry. Peptides corresponding to C9ORF72, WDR41 and SMCR8 are most highly enriched in the motor neuron samples with C9ORF72-long-HA overexpression compared to control samples. (H) The C9ORF72-long-HA expressing embryonic cells were treated with control siRED, siRNA against *WDR41* (si*WDR41*) or siRNA against *SMCR8* (si*SMCR8*) for four days. Protein samples from these cells before and after anti-HA immunoprecipitation were analyzed by western blot for interaction between C9ORF72-long-HA with endogenous proteins as indicated.

**Supplemental figure 2. C9ORF72 long isoform stabilized SMCR8 protein**

(A) A plasmid containing *SMCR8* with C-terminal *GFP*-tag expression cassette was transfected into HEK293 cells alone or in combination with *WDR41-V5, C9ORF72-long-HA* and\or *C9ORF72-short-HA* expression as indicated. GFP intensity was analyzed by flow cytometry as a read-out of SMCR8 protein stability. Representative flow cytometry plot and histogram are shown in the figure for each condition as labeled. (B and C) Percentage of total GFP positive cells (B) and GFP high cells, defined as GFP intensity above 5000 arbitrary unit (C), by flow cytometry analysis of the populations as indicated. N=2 independent experiments; *: p<0.05 by t-test. Error bar represents standard deviation. (D) Representative image of HEK293 cells transfected with the indicated combination of recombinant protein constructs. Arrows are pointing at weak GFP positive cells

**Supplemental figure 3. *Smcr8* mutant mice had enlarged cervical lymph node, splenomegaly and immune cell infiltration into multiple organs**

(A) Additional age-matched control, *C9orf72* -/- and *Smcr8* CRISPR F0 mice were analyzed, showing that both *C9orf72* -/- and *Smcr8* CRISPR mice had significantly enlarged cervical lymph node and spleen. Left panel: representative images of the cervical lymph node and spleen of mice with indicated genotype; right panel: spleen weight of mice with indicated genotype as shown in the left panel. n=4 per genotype, *: p<0.05; **: p<0.01 by one-way ANOVA. (B) Representative image of H&E staining of lung sections from age-matched wild type control and *Smcr8* CRISPR F0 mice (mouse number 31 and 32) showing aggravated immune cell infiltration in the lung of mouse #31. Scale bar: 500um. (C-E) Additional images from H&E staining of spleen (C), liver (D) and lung (E) of 250-day old littermate *Smcr8* +/+ and *Smcr8* -/- mice. Each image represents one individual mouse. Scale bar: 500um. Arrows are pointing at sites of lymphocyte infiltration.

**Supplemental figure 4. Plasma IgG auto-antibody concentration**

Heat-map of plasma IgG activity against 124 different auto-antigens in age-matched *C9orf72* +/+ (n=3), +/- (n=4), -/- (n=2) and *Smcr8* CRISPR F0 (n=15) mice.

**Supplemental figure 5. Additional molecular characterization of *Smcr8* F2 cohort of mice**

(A) Heat-map of plasma concentration of IgG autoantibodies against 124 different auto-antigens in *Smcr8* +/+, +/- and -/- mice, n=5 for each genotype. Unsupervised clustering is shown on the left of the figure. (B) Plasma concentration of a panel of 36 cytokines in 250-day old *Smcr8* +/+ (n=6), +/- (n=6) and -/- (n=5) mice. (C and D) p62 protein expression in the spleen from two independent *Smcr8* F2 cohort of mice, between 50-80 days of age. Upper panel: representative western blot image for endogenous p62 and Gapdh as loading control. Lower panel: quantification of normalized p62 expression relative to its expression in *Smcr8* +/+ samples. (For C: *Smcr8* +/+: n=7, *Smcr8* +/-: n=6, *Smcr8* -/-: n=7; and for D: *Smcr8* +/+: n=3, *Smcr8* +/-: n=3, *Smcr8* -/-: n=4. n.s.: not significant; *: p<0.05; **: p<0.01 by one-way ANOVA. Error bar represents standard deviation).

**Supplemental figure 6. Smcr8 -/- mice developed anemia and neutrophilia**

*Smcr8* +/+ (n=6); +/- (n=7) and -/- (n=5) mice were repeatedly bled every 50 days since 50-day of age until 250-day of age. The peripheral blood samples were analyzed with complete blood cell counts. The following cell counts are shown: (A) Red blood cell count; (B) hemoglobin concentration; (C) Hematocrit; (D) Mean corpuscular volume; (E) Mean corpuscular hemoglobin; (F) Red blood cell distribution width; (G) Platelet count; (H) White blood cell count; (I) Lymphocyte count; (J) Lymphocyte percentage; (K) Neutrophil count; and (L) Neutrophil percentage. *: p<0.05; **: p<0.01; ***: p<0.001, ****: p<0.0001 by Two-way ANOVA with Dunnett’s multiple comparison test. Error bar represents standard deviation.
