# Supplementary figures and images for: The C9orf72-interacting protein Smcr8 is a negative regulator of autoimmunity and lysosomal exocytosis
Source: Genes Dev. 2018 Jul 1;32(13-14):929–43. doi: 10.1101/gad.313932.118 (PMC6075033; doi:10.1101/gad.313932.118)

# GENESDEV/2018/313932\_Zhang et al.\_Supplemental figure 1

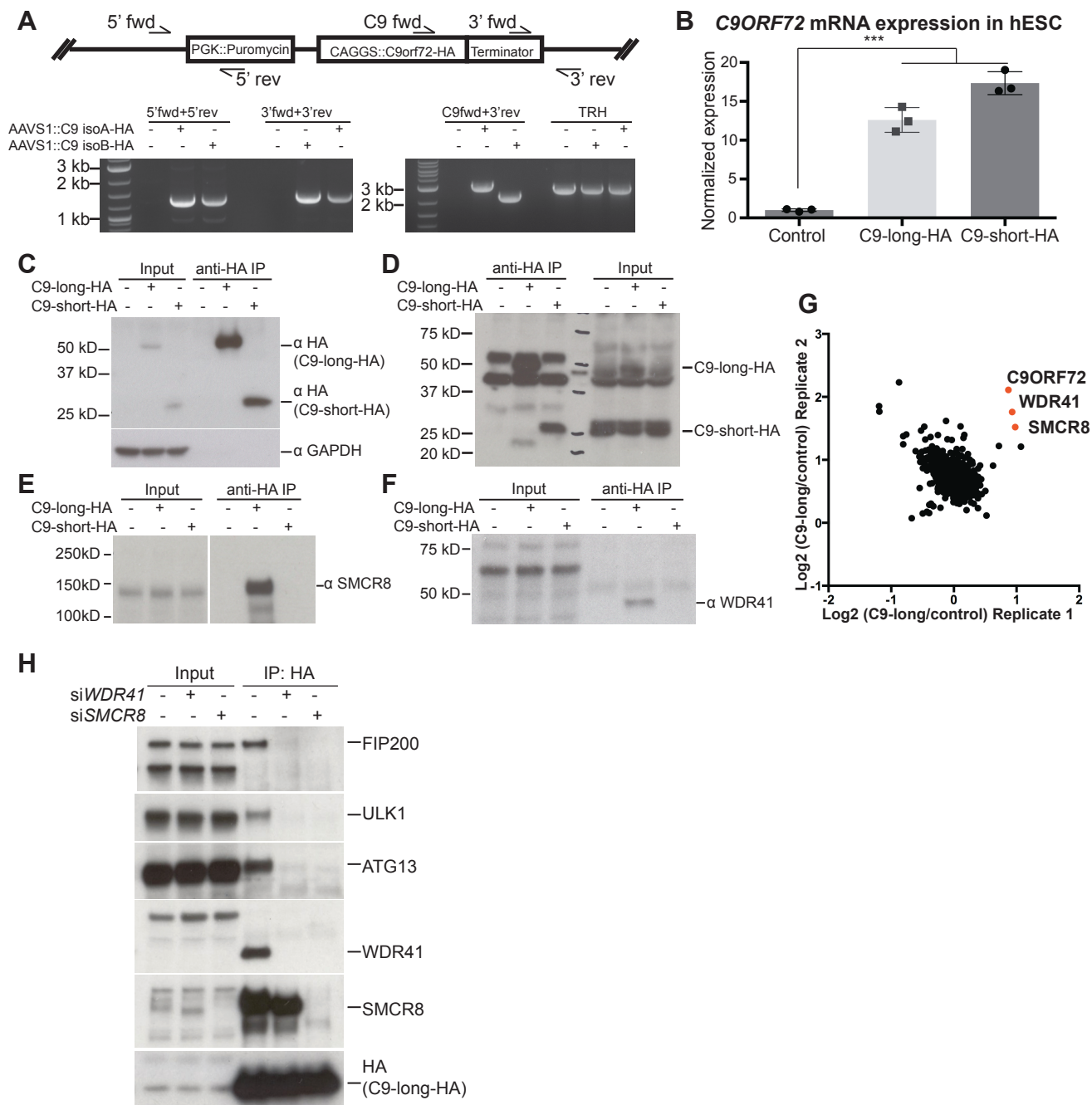

Supplement: Supplemental Material [file supp_gad.313932.118_Supplemental_Fig_S1.ps]

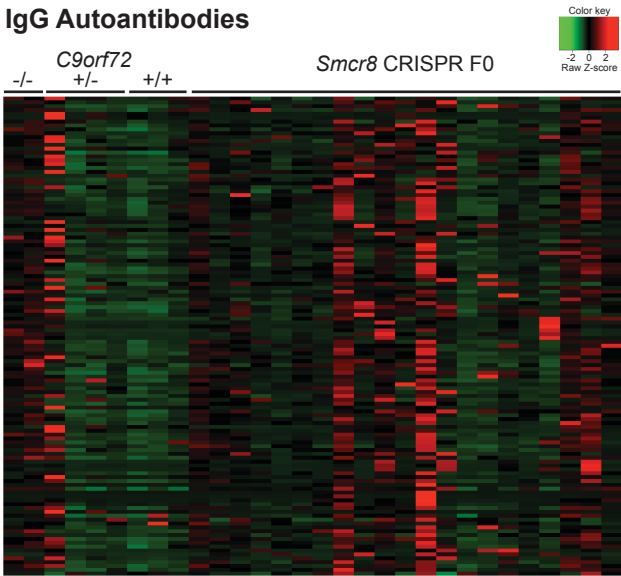

Supplement: Supplemental Material [file supp_gad.313932.118_Supplemental_Fig_S4.ps]

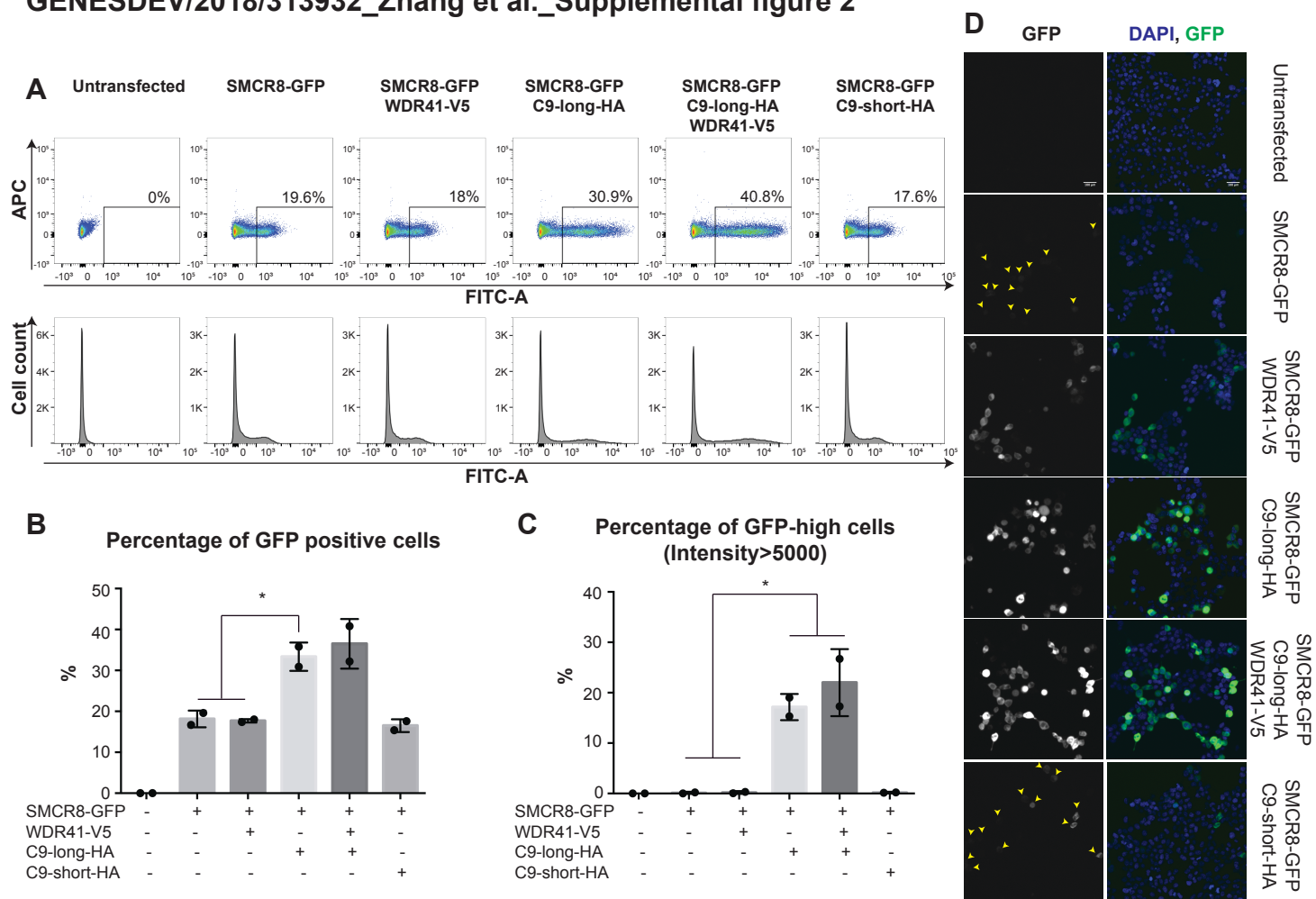

Supplement: Supplemental Material [file supp_gad.313932.118_Supplemental_Fig_S2.ps]

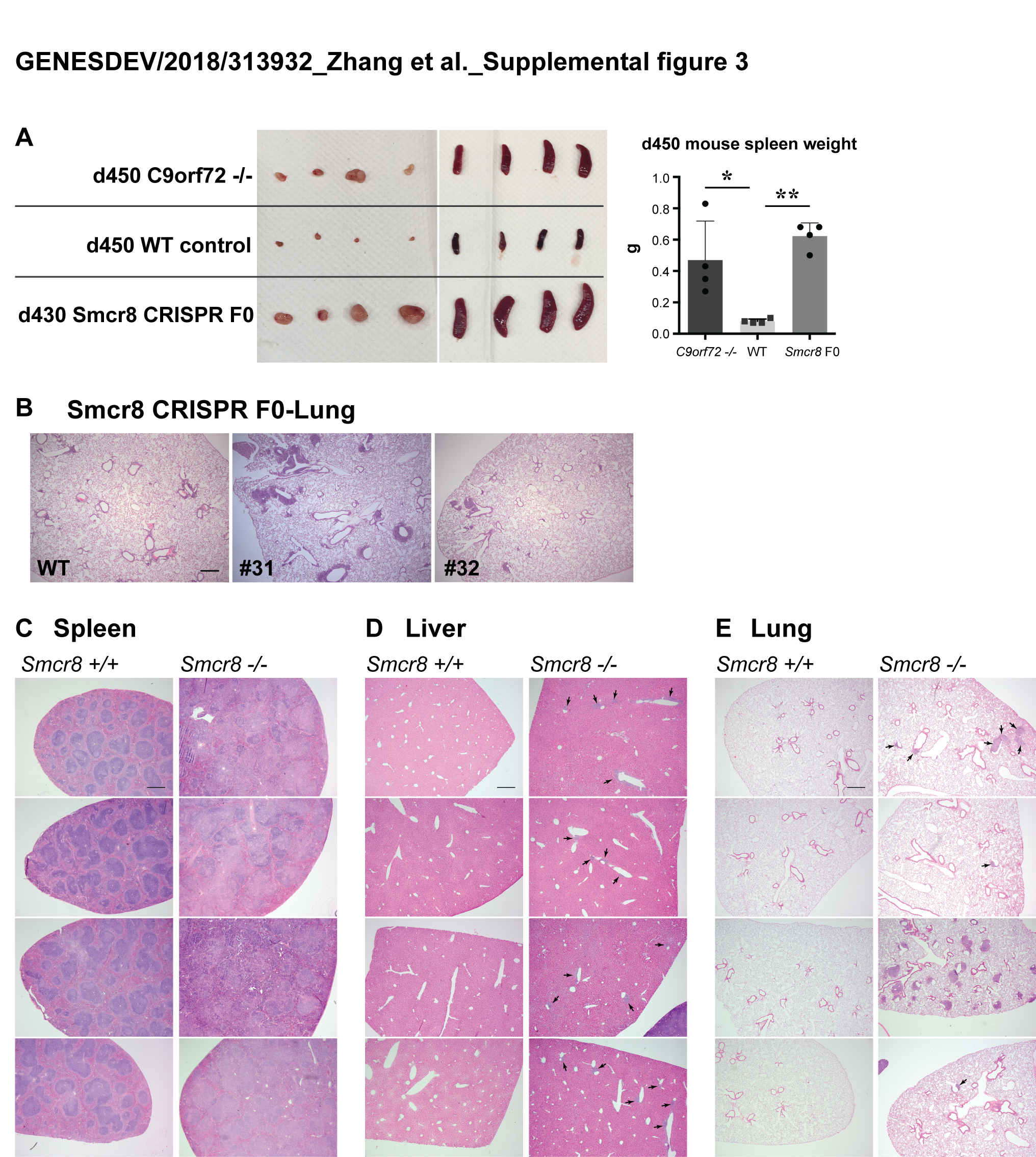

Supplement: Supplemental Material [file supp_gad.313932.118_Supplemental_Fig_S3.tif]

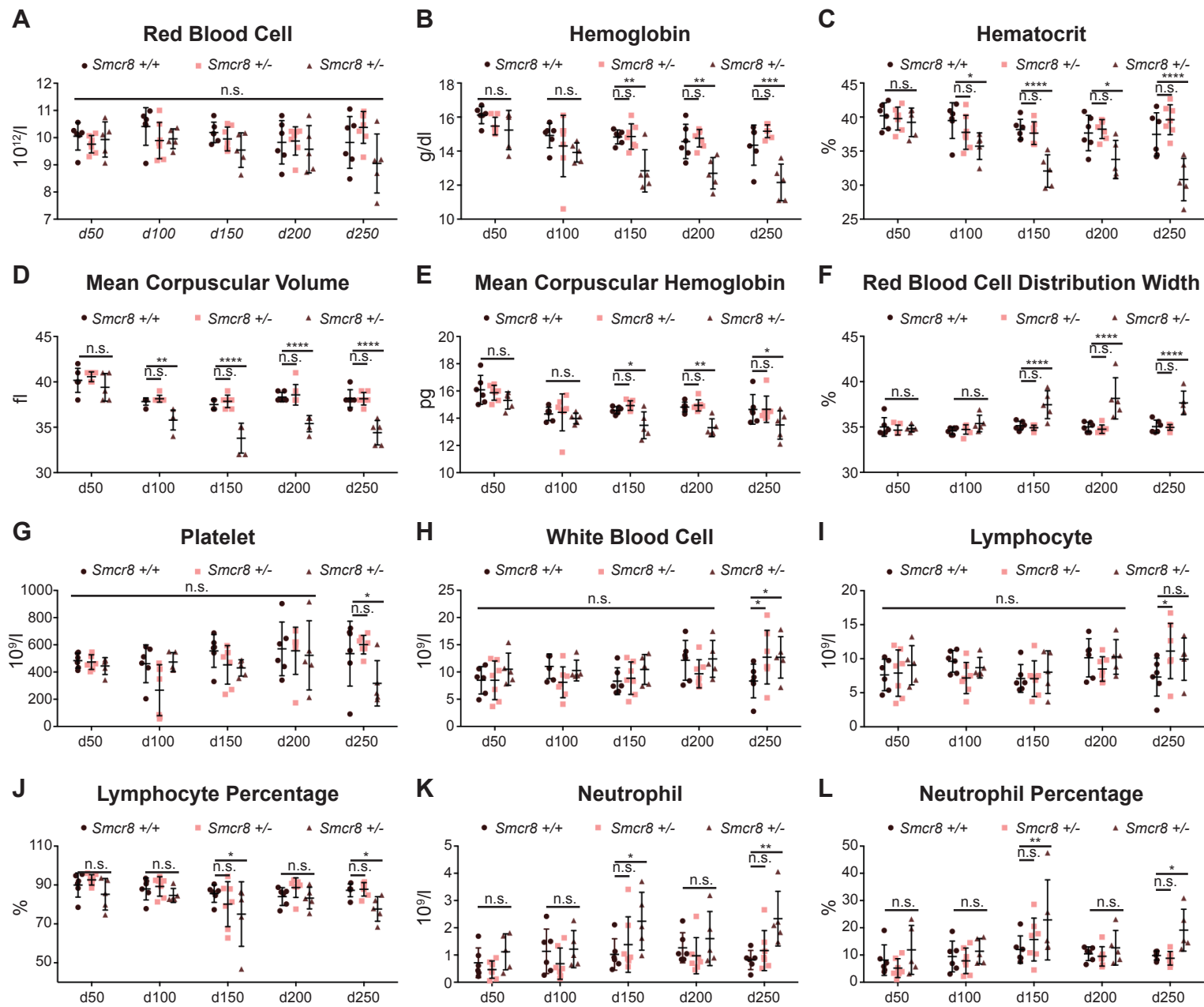

Supplement: Supplemental Material [file supp_gad.313932.118_Supplemental_Fig_S6.ps]
